# Evolution of *cis*-regulatory elements in yeast de novo and duplicated new genes

**DOI:** 10.1186/1471-2164-13-717

**Published:** 2012-12-21

**Authors:** Zing Tsung-Yeh Tsai, Huai-Kuang Tsai, Jen-Hao Cheng, Chih-Hsu Lin, Yuan-Fan Tsai, Daryi Wang

**Affiliations:** 1 Bioinformatics Program, Taiwan International Graduate Program, Academia Sinica, Taipei, 115, Taiwan; 2 Institute of Information Science, Academia Sinica, Taipei, 115, Taiwan; 3 Institute of Biomedical Informatics, National Yang-Ming University, Taipei, 112, Taiwan; 4 Research Center for Information Technology Innovation, Academia Sinica, Taipei, 115, Taiwan; 5 Biodiversity Research Center, Academia Sinica, Taipei, 115, Taiwan; 6 Department of Social and Regional Development, National Taipei University of Education, Taipei, 106, Taiwan

**Keywords:** De novo gene, Regulatory evolution, TFBS turnover, Promoter architecture

## Abstract

**Background:**

New genes that originate from non-coding DNA rather than being duplicated from parent genes are called de novo genes. Their short evolution time and lack of parent genes provide a chance to study the evolution of *cis*-regulatory elements in the initial stage of gene emergence. Although a few reports have discussed *cis*-regulatory elements in new genes, knowledge of the characteristics of these elements in de novo genes is lacking. Here, we conducted a comprehensive investigation to depict the emergence and establishment of *cis*-regulatory elements in de novo yeast genes.

**Results:**

In a genome-wide investigation, we found that the number of transcription factor binding sites (TFBSs) in de novo genes of *S*. *cerevisiae* increased rapidly and quickly became comparable to the number of TFBSs in established genes. This phenomenon might have resulted from certain characteristics of de novo genes; namely, a relatively frequent gain of TFBSs, an unexpectedly high number of preexisting TFBSs, or lower selection pressure in the promoter regions of the de novo genes. Furthermore, we identified differences in the promoter architecture between de novo genes and duplicated new genes, suggesting that distinct regulatory strategies might be employed by genes of different origin. Finally, our functional analyses of the yeast de novo genes revealed that they might be related to reproduction.

**Conclusions:**

Our observations showed that de novo genes and duplicated new genes possess mutually distinct regulatory characteristics, implying that these two types of genes might have different roles in evolution.

## Background

New genes arise through various mechanisms, including gene duplication, exon shuffling, gene fusion, retroposition, mobile elements, lateral gene transfer, and de novo origination [1-3]. Although new genes are considered to be fairly dispensable [4], their role in adaptive evolutionary innovation has been investigated. Most of the studies have focused on the cellular, physiological, morphological, behavioral, and reproductive phenotypic traits associated with new genes [1,5-7]. A recent study found that 30% of the new genes in *Drosophila* quickly evolved essential functions that allowed them to participate in development [8]. Using pre-existing genes as the raw material, duplicate genes rapidly developed essential functions that were not present in the pre-duplication gene through the processes of neofunctionalization [8] or subfunctionalization [9]. In addition, neofunctionalization and subfunctionalization of transcription factor binding sites (TFBSs) can explain the novelty that occurs in the regulatory region of duplicated new genes [10-12]. The de novo origin of genes, genes that arise from previous nonfunctional genomic sequences, is a rare and intriguing process [13,14]. It is believed that the new coding region could emerge by mutations that remove disruptions of a proto-open reading frames [1]. Positive selection in the coding sequences has been reported, suggesting that adaptive protein evolution had occurred [15].

De novo gene evolution was first investigated in *Drosophila melanogaster* in 2006. Five novel genes were identified experimentally as derived from ancestral non-coding sequences and evolved as the result of a selection process associated with male reproduction [16]. In *Saccharomyces cerevisiae*, the first identified de novo gene was *BSC4*. Population genetic analysis suggested that *BSC4* was under strong negative selection at the nonsynonymous sites [3]. A de novo transcript in *Mus musculus* was found to have emerged in an intergenic region because of indel mutations in the 5’ regulatory region; the transcript was fixed by a selective sweep in *M*. *musculus* populations [17]. Other de novo genes have been identified in various species; for example, *CLLU1* and *FLJ33706* in *Homo sapiens*[18,19], *MDF1* in *S*. *cerevisiae*[20], *DR10* in *Oryza sativa*[21], and *Noble* in *D*. *melanogaster*[22]. In addition, several genome-wide analysis studies have identified numerous de novo genes in various species, and the importance of such genes in adaptive evolution has been discussed [23-28]. For example, in *D*. *melanogaster*, a study based on expressed sequence tags identified eleven putative de novo genes, and de novo origination was estimated to be responsible for 11.9% of the new genes [24]. In *H*. *sapiens*, 60 protein-coding genes were identified as de novo genes that were highly expressed in the cerebral cortex [27]. These findings indicate the importance of de novo genes in phenotypic diversity and evolutionary adaptation. Nevertheless, the regulatory evolution of de novo genes is not yet fully understood. A prevalent view is that de novo genes do not possess complicated regulatory control and, therefore, only a functional transcription start site would be required for transcription initiation [29]. However, because de novo genes might play important roles in development [8], the view that only a simple regulatory control mechanism is used remains open to speculation.

Several genome-wide studies have attempted to describe the characteristics of regulatory evolution [30,31]. Frequent gain or loss events of TFBSs (TFBS turnover) have been identified as an important feature of regulatory evolution, and have been found to exhibit lineage specificity in transcriptional regulation [32-34]. A previous study showed that duplicated new genes inherit more than a third of the regulatory interactions from their ancestral genes [35]. Moreover, the expression of duplicated genes often benefits from the preexisting regulatory mechanism [36]. After gene duplication, positive selection on *cis*-regulatory motifs leading to dramatically accelerated rates of *cis*-regulation compared with the orthologs has been observed [37]. In *S*. *cerevisiae*, it has been shown that the number of shared TFBSs in duplicate genes decreased with evolution time whereas the total number remained unchanged, suggesting that there is a balance between gain in functionally novel TFBSs and either the loss of preexisting TFBSs or the modification of preexisting TFBSs to new functions [12]. Nonetheless, de novo genes evolve from non-coding sequences based on the cryptic presence of functional sites, including a transcriptional start site and upstream regulatory elements [29]. The question of how de novo genes that have no parent gene obtain regulatory elements and further establish complex regulatory mechanisms has yet to be determined.

We conducted a genome-wide investigation of de novo genes in *S*. *cerevisiae* to investigate regulatory evolution in the initial stages of gene emergence. One of the challenges is that the conventional methods that are used for de novo gene identification are known to overestimate their numbers because of the high number of false positives that are generated [27]. Recently, Capra et al. developed a computational pipeline to identify de novo genes in yeast and to understand the evolution of protein interaction networks involving the novel genes [38]. They identified 227 de novo genes that originated after whole-genome duplication (WGD), and found that initially the de novo genes had fewer interactions, but subsequently gained interactions more rapidly than duplicated new genes. Here, we modified their pipeline to identify *S*. *cerevisiae*-specific de novo genes that emerged after divergence from *S*. *paradoxus*, instead of after WGD. The stringent criteria that we used to identify de novo genes would aid our observation of *cis*-regulatory element evolution during the initial stage of a gene emergence. Using our modified method, we identified 34 de novo genes that were specific to *S*. *cerevisiae* (i.e., without either paralogous genes or orthologous genes in any other species). To analyze the *cis*-regulatory evolution of genes that had emerged from different origins and had different ages, we identified duplicated new genes (new genes with paralogous genes) and orthologous genes (well-conserved genes with orthologous genes in all seven yeast species) and compared the characteristics of *cis*-regulation in each. We found a higher number of TFBS gain events and higher evolution rates in the promoters of new genes (both de novo and duplicated new genes) compared with in old (orthologous) genes. Our findings suggested that the promoters of new genes might experience adaptive evolution as their functions become established. Furthermore, we investigated the nucleosome architecture in the promoter regions, which might be associated with transcriptional regulation and the evolution of eukaryotic genes [39-46]. Our results revealed significant lower occupancy of proximal nucleosomes and lower enrichment of the TATA box in promoters of de novo genes compared with in duplicated new and orthologous genes, suggesting that de novo genes might employ different regulatory strategies from duplicated genes. Finally, functional analyses revealed that de novo genes might play roles in reproduction-related functions.

## Methods

### Identification of de novo genes in *S*. *cerevisiae*

**Figure 1:**
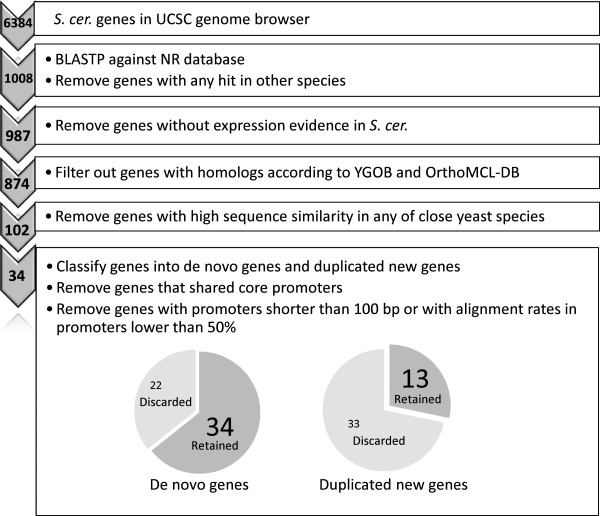
**The computational pipeline for identifying de novo genes and duplicated new genes in *S. cerevisiae. ***All the *S. cerevisiae* genes that have BLASTP hits only in *S. cerevisiae* were collected. Genes with possible homologs were excluded accordingly. The remaining genes were further classified into de novo genes and duplicated new genes. Last, genes with short promoters, poor alignments in promoters, or share core promoters were excluded. Finally, 34 de novo genes and 13 duplicated new genes were selected for subsequent analyses in this study. The numbers on the left denotes the number of remaining genes in each step. The pie chart illustrates the proportion of genes used in our analysis. The darker parts (34 and 13) are the number of genes retained, and the lighter parts (22 and 33) are the number of genes discarded.

The *S*. *cerevisiae* genome assembly sequence (SacCer_Apr2011/sacCer3) and genome annotation from the Saccharomyces Genome Database (SGD) [47] were used. The protein sequences of 6,384 *S*. *cerevisiae* genes were downloaded from UniProt [48]. These sequences were compared against the NCBI non-redundant protein sequence database (NR) using BLASTP with an e-value cut-off of 10^-10^. We identified 1,008 UniProt sequences with no BLASTP hits to proteins in other species and at least one hit in *S*. *cerevisiae*. We selected 987 genes that had expression evidence in a high-resolution transcriptome map [49] from the 1,008 genes. To ensure that the 987 genes had no known homologous genes, we queried two additional databases, Yeast Gene Order Browser (YGOB version 6) [50] and OrthoMCL-DB (version 5) [51]. YGOB curates homology information by gene synteny with the manual reconstruction of the duplication history in the recent evolution of the *S*. *sensu stricto* and *S*. *sensu lato* yeasts [50]. OrthoMCL-DB houses ortholog group predictions for 150 species (version 5), and querying this database would avoid the possibility of including horizontal transfer genes in our study [51]. After removing the genes with homologs that were found in YGOB or OrthoMCL-DB, the 874 genes that remained were identified as *S*. *cerevisiae*-specific new genes, which had no annotated homologous genes in other species. We would like to have a conservative approach, so we further filtered out potential orthologous genes with high nucleotide sequence identity (>70%) to any one of the six closely related yeast species (*S*. *paradoxus*, *S*. *mikatae*, *S*. *kudriavzevii*, *S*. *bayanus*, *S*. *castelli* and *S*. *kluyveri*). Sequence identity was calculated from the UCSC multiz alignment of seven yeast species (multiz7way) [52]. After applying this approach, 102 new candidate *S*. *cerevisiae*-specific genes that had emerged in *S*. *cerevisiae* after its divergence from *S*. *paradoxus* were identified. Next, we classified the 102 new genes according to their origins into 56 de novo genes and 46 duplicated new genes; new genes with paralogous genes in *S*. *cerevisiae* (based on a list of 1,048 independent duplicate pairs [39]) were defined as duplicated new genes, and new genes without paralogous genes were defined as de novo genes. To minimize potential biases, we also removed three types of genes based on the characteristics of the promoter regions, which were defined as the intergenic regions (≤1000 bp) upstream of the transcriptional start sites (TSSs). The three types of genes that were excluded from the subsequent analyses were as follows: (a) genes with promoters shorter than 100 bp were removed because such promoters were not long enough for us to be able to analyze nucleosome occupancy in TSS-distal region; (b) head-to-head genes that shared core promoters (250 bp upstream of the TSS) were removed because the promoter of one of the genes in a head-to-head pair may already contain the TFBSs of the other gene, thereby biasing the estimation of preexisting TFBSs (Only genes that shared core promoters were removed based on the assumption that core promoters contain most of the functional TFBSs); (c) genes with promoters that were poorly aligned against the *S*. *paradoxus* orthologous sequences (a gap rate greater than 50%) were removed because the poor alignments may have biased our estimation of the evolution rate. After applying these filtering criteria, 34 de novo genes and 13 duplicated new genes remained and were used in the subsequent analyses (Figure 1). Additionally, we identified orthologous relationships in the six closely related yeast species for each gene in *S*. *cerevisiae* according to synteny and sequence similarity under the same criteria as above. The genes that have orthologs in all the six yeast species were selected as orthologous genes. All the genes in the three gene categories were listed in Additional file 1: Table S1.

### Identification of transcription factor binding sites

We retrieved 481 position frequency matrices from the MYBS database which integrates ChIP-chip data and phylogenetic footprinting data in yeast [53]. To remove redundant motifs, we integrated all the recorded motifs for each transcription factor (TF) using the STAMP web server which calculates the similarity of various motifs and integrates them into a familial binding profile [54]. A total of 175 familial binding profiles were generated and converted into position weight matrices (PWMs) by the PATSER software using the default settings [55]. Putative TFBSs were obtained by scanning PWMs with a threshold *p*-value of <0.001 [56] (TFBSs identified under different thresholds were also investigated to examine the robustness of our study in the Additional file 2: Supplementary Document). Next, putative TFBSs that were not documented in the curated YEASTRACT database, which documents 48,333 regulatory associations between TFs and their target genes [57], were excluded. We then characterized TFBSs based on whether they were newly gained (i.e., did not exist before gene origination) or were preexisting TFBSs (i.e., already existed before gene origination). The characterization entailed scanning the corresponding regions of *S*. *paradoxus* and *S*. *mikatae*, the two yeast species most closely related to *S*. *cerevisiae*, for each of the TFBSs that were identified in *S*. *cerevisiae*. The corresponding regions, defined as the regions that extended 25 bp upstream and downstream of the aligned region of a TFBS [53], were retrieved from multiz7way [52]. A TFBS gain event was defined as a TFBS in *S*. *cerevisiae* that did not possess an occurrence of its motif within the corresponding regions in *S*. *paradoxus* and *S*. *mikatae*. A preexisting TFBS was defined as possessing occurrences of its motif within the corresponding regions in *S*. *paradoxus*, *S*. *mikatae*, or both. TFBS losses of de novo genes were not investigated because no ancient gene exists; that is, no functional TFBS existed before the de novo gene emerged.

### Investigation of nucleosome occupancy and promoter architecture

In this study, we used a *S*. *cerevisiae* genome-wide reference map of nucleosome positions that integrated six high-resolution genome-wide maps from multiple laboratories and detection platforms [58]. To exclude relatively depleted nucleosomes, only nucleosomes with >50% occupancy were considered [58]. Tirosh et al. defined two gene categories according to different characteristics of the promoter nucleosomes and found that the two categories possessed different regulatory strategies [45]. We modified the procedure proposed by Tirosh et al., and identified two categories according to the presence of nucleosomes in the TSS-proximal region (from TSS up to −100) and the TSS-distal region (from −300 to −400), as follows: (a) genes with a nucleosome in the TSS-proximal region but with none in the TSS-distal region, referred to as occupied proximal nucleosome (OPN) genes; and (b) genes without a nucleosome in the TSS-proximal region but with one in the TSS-distal region, referred to as depleted proximal nucleosome (DPN) genes.

### Functional analysis

The Serial Pattern of Expression Levels Locator (SPELL) database [59] was used to identify the potential functions of the *S*. *cerevisiae* de novo genes. SPELL is a query-driven search engine for large gene expression microarray compendia containing more than 2,400 experimental conditions. It has been used to identify the most informative expression data sets and to interpret relevant genes for a given set of query genes. We queried the SPELL database using the de novo genes and identified the top 100 relevant genes that were most similarly expressed across all data sets. SPELL then assigned the Gene Ontology (GO) terms from the identified genes to the queried de novo genes. Significance was tested using the Bonferroni-corrected Fisher’s exact test with the *q*-value set to <0.01 [59]. We also conducted TFBS enrichment analysis to identify TFs that might be responsible for the regulation of the de novo genes. The identification was based on a binomial test, in which the null hypothesis states that the probability of finding the TFBSs in de novo genes is smaller or equal to that of all the other genes in the *S*. *cerevisiae* genome.

## Results

### Evolutionary characteristics of TFBSs in de novo genes

**Figure 2:**
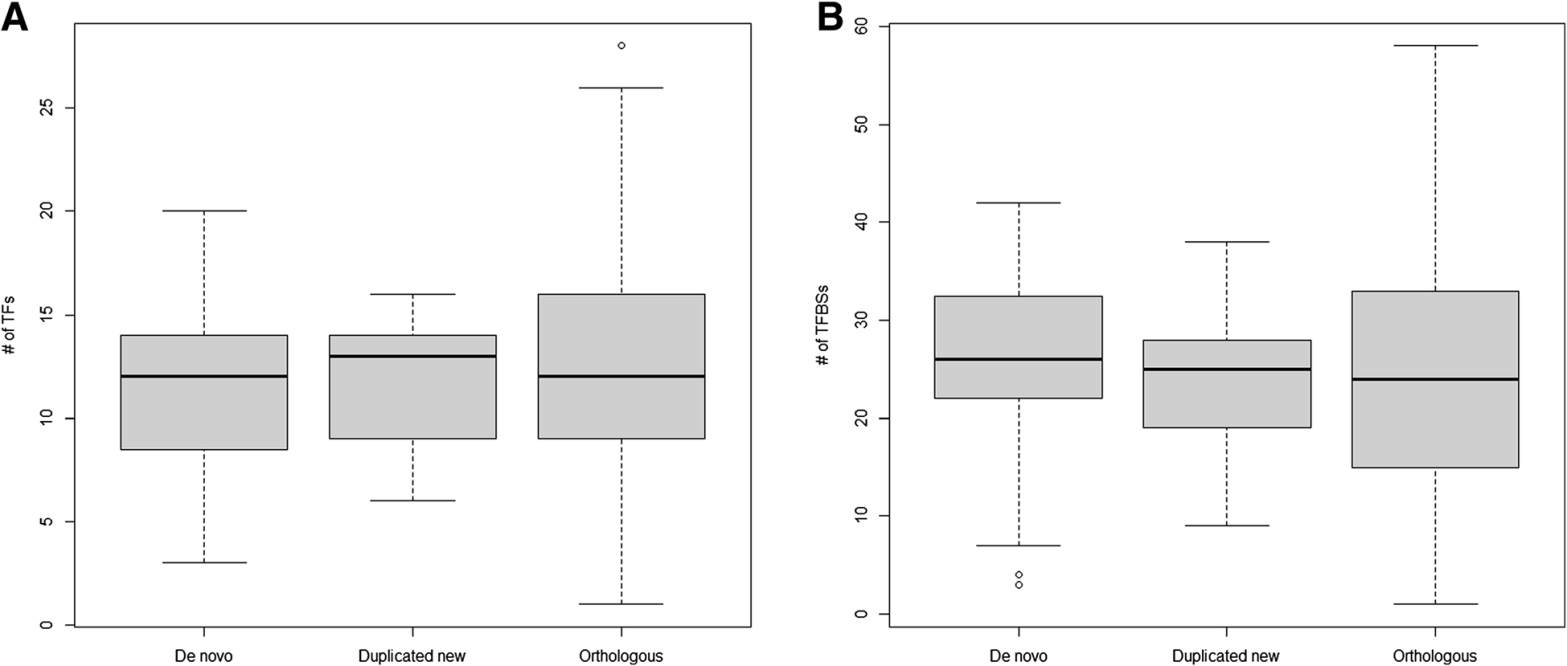
**The numbers of TFs and TFBSs for de novo genes, duplicated new genes, and orthologous genes.** Comparison of the average number of TFs regulating a gene (**A**), and the average number of TFBSs in a gene (**B**) for de novo genes, duplicated new genes, and orthologous genes. No significant differences were observed (two-sided Wilcoxon test).

To investigate whether a promoter was well established before the emergence of a new coding gene or had evolved rapidly after the origination, we analyzed the number and evolutionary characteristics of TFBSs in the promoters of the three gene groups. On average, a de novo gene was regulated by 11.5 ± 0.8 TFs, a duplicated new gene was regulated by 11.7 ± 0.6 TFs, and an orthologous gene was regulated by 11.9 ± 0.3 TFs. The average number of TFBSs identified in a promoter was 25.4 ± 1.9 for de novo genes, 24.4 ± 1.6 for duplicated new genes, and 24.3 ± 0.6 for orthologous genes. The average numbers of TFs regulating a gene and the average number of TFBSs in a promoter were similar in the new genes (de novo or duplicated new) and old genes (orthologous) (Figure 2).

**Figure 3:**
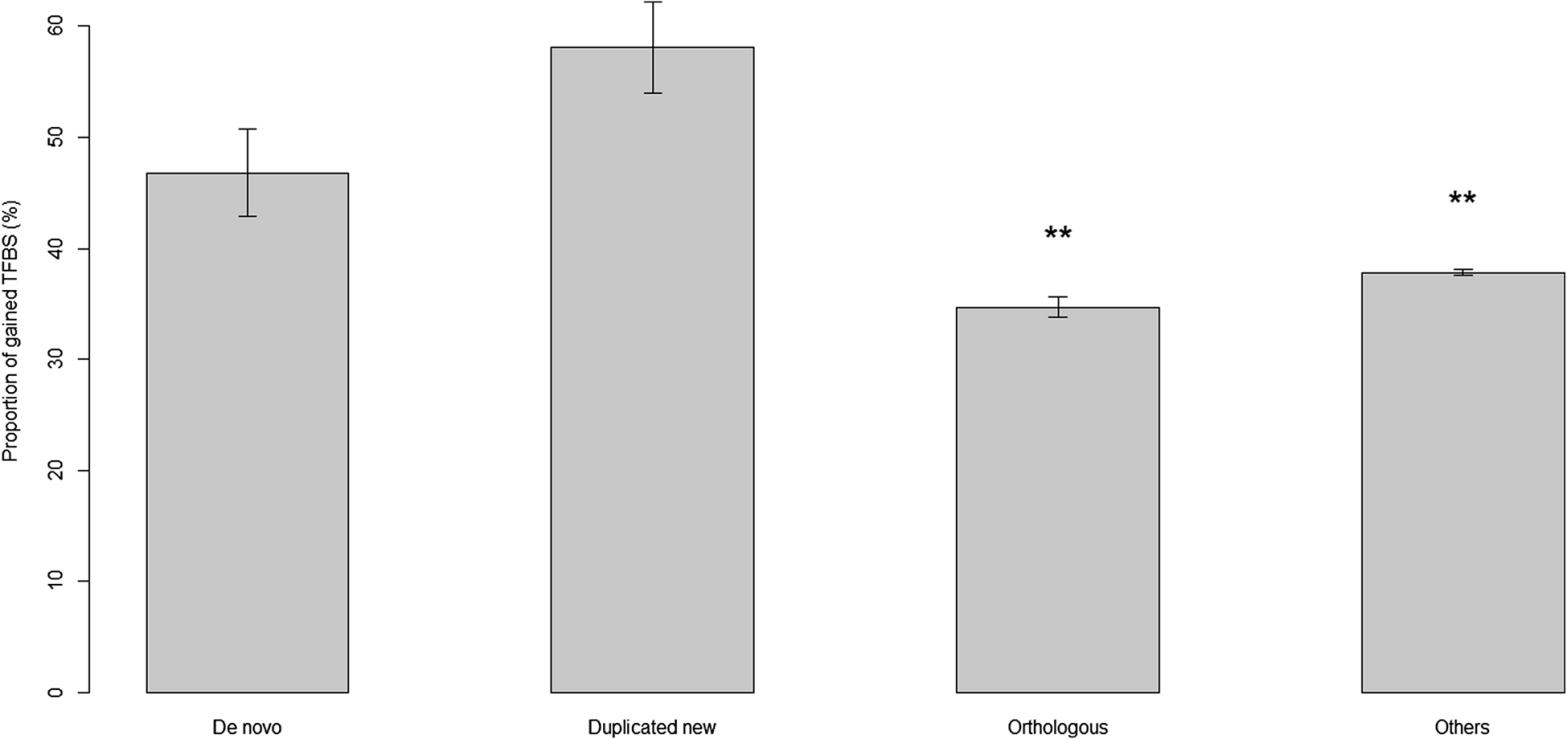
**The proportions of gained TFBSs in de novo genes, duplicated new genes, orthologous genes, and all the other genes in the *S. cerevisiae genome. ***Comparisons of the proportions of gained TFBSs in de novo genes, duplicated new genes, orthologous genes, and all the other genes in the *S. cerevisiae* genome. The significance tests were conducted by one-sided Wilcoxon test (*: *p*-value < 0.01; **: *p*-value < 0.001 compared with de novo genes).

To examine the origination of the TFBSs in de novo and duplicated new genes, we classified TFBSs into newly gained TFBSs, and preexisting TFBSs. Significantly higher proportions of gained TFBSs were observed for new genes (de novo and duplicated new) compared with the orthologous genes (one-sided Wilcoxon test *p* = 7.3×10^-4^ and *p* = 2.7×10^-7^, respectively) and all the other genes in the *S*. *cerevisiae* genome (Figure 3). The difference between gained TFBSs in de novo and duplicated new genes was not significant (two-sided Wilcoxon test *p* = 0.06). On average, 53.2% and 41.9 % of the TFBSs were found to be preexisting TFBSs in the promoters of de novo genes and duplicated new genes, respectively; and 65.3% and 62.2% of the TFBSs were preexisting in the promoters of orthologous genes and all the other genes in the *S*. *cerevisiae* genome, respectively. The proportions of preexisting TFBSs were significantly smaller in both the de novo and duplicated new genes compared with in the orthologous genes (one-sided two-sample proportion test *p* = 3.5×10^-11^ and *p* = 2.2×10^-16^, respectively). No statistically significant difference was found between the two types of new genes (two-sided two-sample proportion test *p* = 0.043). Because the new and old genes possessed similar numbers of regulatory elements (Figure 2), we inferred that the *cis*-regulatory elements of new genes evolved rapidly after the emergences of the genes. Although only duplicated new genes originated from the copying of ancestral functional sequences, both types of new genes shared similar benefits from the preexisting TFBSs.

### Lower selection pressure in promoter regions of de novo genes

**Figure 4:**
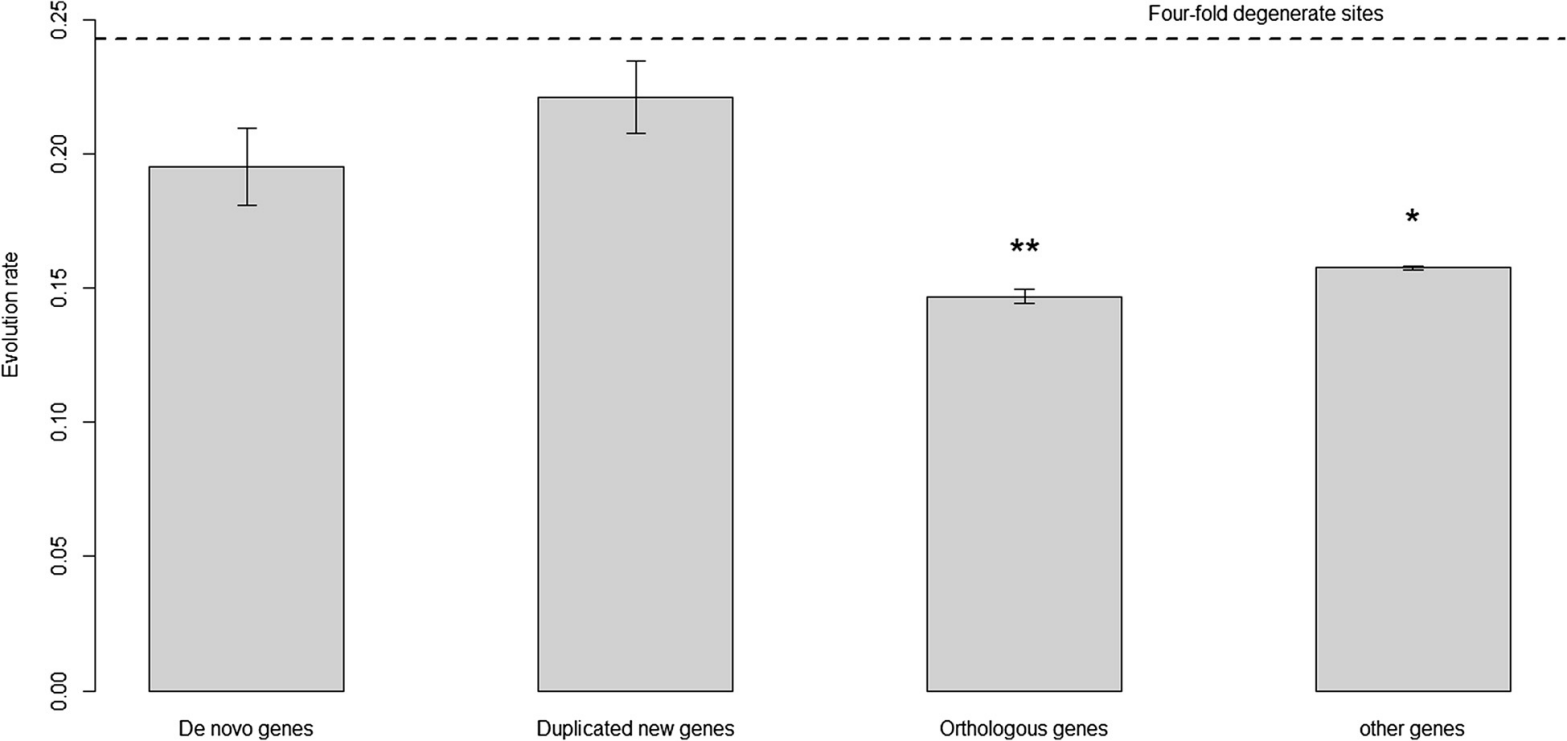
**The promoter evolution rates of de novo genes, duplicated new genes, orthologous genes, and all the other genes in the *S. cerevisiae *genome.** Comparisons of evolution rates in the promoters of de novo genes, duplicated new genes, orthologous genes, and all the other genes in the *S*. *cerevisiae* genome. The dashed line indicates the evolution rate of four-fold degenerate sites. The significance tests were conducted by one-sided Wilcoxon test (*: *p*-value < 0.01; **: *p*-value < 0.001 compared to de novo genes)

To investigate the differences in selection constraint among de novo genes, duplicated new genes, and orthologous genes, we determined the DNA substitution rate as an evolution rate for the promoters of genes in each group (Figure 4). The DNA substitution rate for each promoter region was calculated from pairwise alignments between the *S*. *cerevisiae* and *S*. *paradoxus* promoter sequences without considering indels. The evolution rate in promoter sequences of de novo genes was significantly higher than in orthologous genes and all the other genes in the *S*. *cerevisiae* genome (one-sided Wilcoxon test *p* = 1.6×10^-4^ and *p* = 1.7×10^-3^, respectively). To further verify the selection pressure, we compared the evolution rates that we obtained with those of the four-fold degenerate sites that were suggested as neutral references in yeast [60]. 1,952,398 four-fold degenerate sites were identified in 6,392 yeast genes. The result of the comparison showed that the evolution rates in the promoters of the three gene groups were lower than in the neutral references, indicating that the promoters were under selection pressure. However, the promoters of de novo genes might experience lower selection pressure than the promoters of orthologous genes. Similar results were found for duplicated new genes, and no significant difference was observed in promoter evolution rates between the two groups of new genes (two-sided Wilcoxon test *p* = 0.14).

### Nucleosome occupancy and TATA box in promoters of new genes

**Figure 5:**
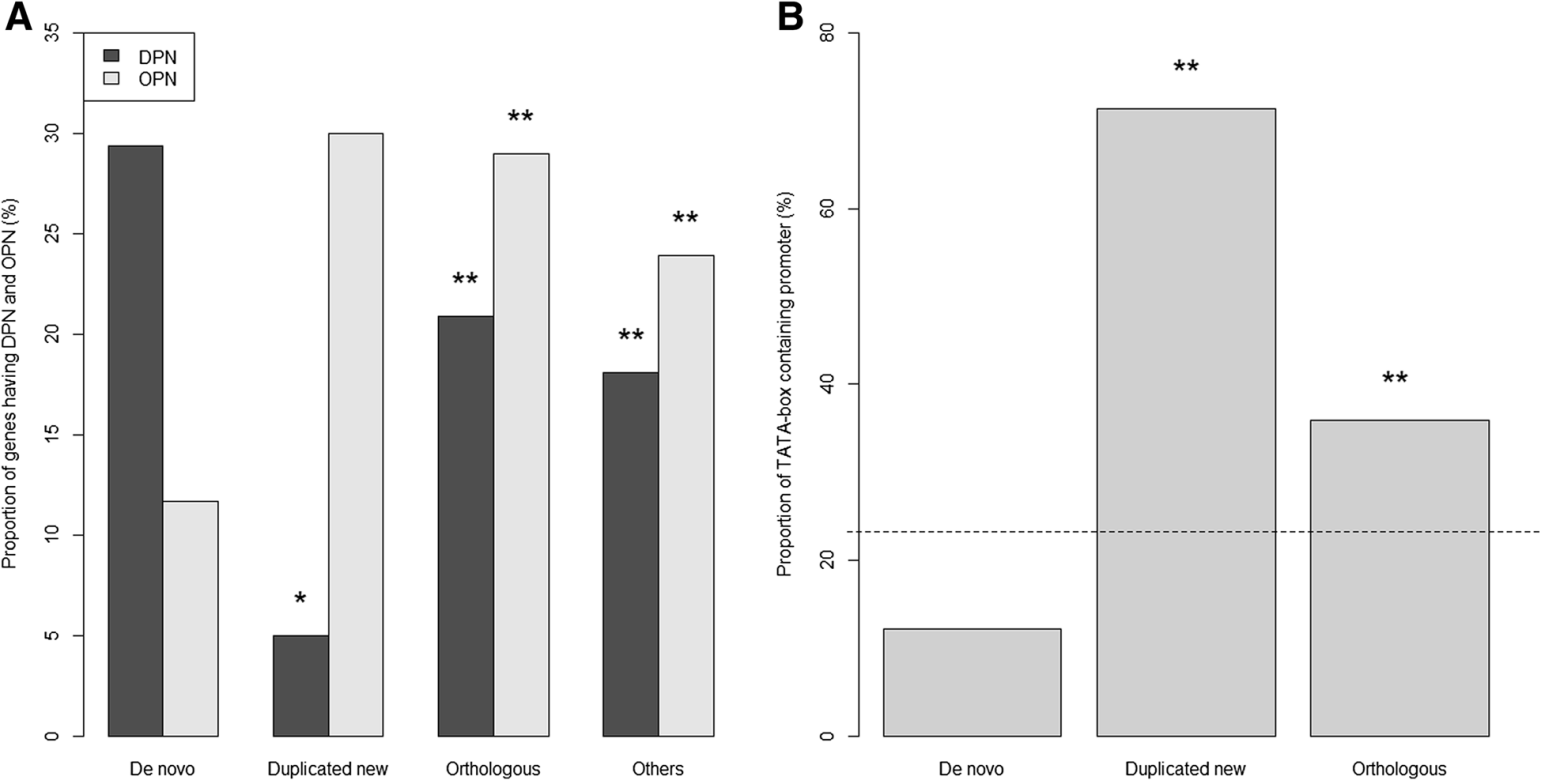
**The nucleosome occupancy and TATA box of de novo genes, duplicated new genes, orthologous genes, and all the other genes in the *S. cerevisiae *genome.** Proportions of (**A**) DPN and OPN genes and (**B**) TATA box-containing genes in de novo genes, duplicated new genes, orthologous genes, and all the other genes in the *S*. *cerevisiae* genome. The dashed line indicates the proportion of TATA box-containing genes in the whole genome. The significance tests were conducted by one-sided two-sample proportion test (*: *p*-value < 0.01; **: *p*-value < 0.001 compared with de novo genes).

Previous studies have shown that nucleosomes may participate in transcriptional regulation and that the sequences occupied by nucleosomes are subjected to various evolutionary constraints [39-43]. To investigate the effect of nucleosome architecture on the regulatory evolution of de novo genes, we analyzed the nucleosome occupancy in the promoters of de novo versus duplicated new and orthologous genes. The comparisons were based on the findings of Tirosh et al. who suggested that the presence of nucleosomes in the TSS-proximal region might be related to high transcriptional plasticity and might be associated with evolvability of a gene [45,61]. Our results showed that DPN genes (genes with nucleosome depleted in TSS-proximal regions) were significantly predominant in de novo genes (one-sided two-sample proportion test *p* = 0.002), whereas OPN genes (genes with nucleosome occupied in TSS-proximal regions) predominated in duplicated new genes and orthologous genes (one-sided two-sample proportion test *p* = 3.5×10^-5^ and *p* = 2.8×10^-4^) (Figure 5A). Moreover, compared with all the other genes in the *S*. *cerevisiae* genome, the proportion of DPN genes in the de novo genes was also significantly higher (one-sided two-sample proportion test *p* = 0.003). Conversely, the proportion of DPN genes in duplicated new genes was significantly lower than in all the other genes in the *S*. *cerevisiae* genome (one-sided two-sample proportion test *p* = 0.01).

Another crucial architectural motif in the promoter is the TATA box. The expression of TATA-containing genes is highly regulated, responsive to stress, sensitive to chromatin regulators, and variable across different species [62,63]. We found that the proportion of TATA-containing genes (consensus TATA(A/T)A(A/T)(A/G) within −50 to −200 [63]) was significantly lower in de novo genes (12.1%) compared with the proportion observed in the whole *S*. *cerevisiae* genome (23.3%) (one-sided two-sample proportion test *p* = 0.0037). In contrast, the proportion of TATA-containing genes in the duplicated new genes (71.4%) and orthologous genes (35.9%) was significantly higher than in the whole *S*. *cerevisiae* genome (one-sided two-sample proportion test *p* = 3.5×10^-7^ and 1.1×10^-9^, respectively) (Figure 5B). Overall, our findings indicated that de novo genes were dominated by DPN genes but fewer TATA-containing genes, whereas duplicated new genes were dominated by OPN genes and TATA-containing genes. These results suggested that the two types of new genes may possess different regulatory strategies.

### Functional analyses of de novo genes

To investigate the possible roles of de novo genes, we conducted a number of analyses to infer their potential functions. We used FunSpec [64], a web-based cluster interpreter that identifies enriched function annotations across numerous knowledge sources. As expected, because the de novo genes were lack of orthologous genes, most de novo genes were functionally unknown. Therefore, we used SPELL (Version 2.0.3) [59] to identify the informative expression and potentially related GO terms of the identified de novo genes. Twenty-two GO terms with corrected *q*-values <0.01 were suggested as potential functional annotations for these genes (Table 1). Most of the 22 GO terms are related with reproduction, including formation of sporulation, spore, ascospore, and cell differentiation. When we included the 22 de novo genes that were excluded from the previous analyses because of short or poorly aligned promoters, SPELL again predicted that the 56 unfiltered de novo genes could have functions related to reproduction (Table 2). The GO terms, meiosis and meiotic cell cycle, are also enriched in the de novo genes. These analyses suggested that de novo genes might play roles in reproduction. We were curious about whether the TFs that were enriched in the regulated de novo genes also had functions related to reproduction. The TFBS enrichment analysis indicated that three TFs, BAS1, GCN4 and GCR1, might be responsible for the regulation of de novo genes (binomial test *q*-value <0.001). BAS1, GCN1, and GCR1 are known to play important roles in meiotic recombination in reproduction processes [65-68], which coincides with the functional annotation of the de novo genes. The above results suggested that de novo genes may be regulated by reproduction-related TFs and involved in reproduction.

**Table 1:** **Predicted GO terms for 34 de novo genes by SPELL**[59]

**GO term**	***q-value***
sexual sporulation	2.1×10^-3^
sexual sporulation resulting in formation of a cellular spore	2.1×10^-3^
cellular process involved in reproduction	3.8×10^-3^
reproductive process	3.8×10^-3^
spore wall biogenesis	4.3×10^-3^
ascospore wall biogenesis	4.3×10^-3^
ascospore wall assembly	4.3×10^-3^
spore wall assembly	4.3×10^-3^
fungal-type cell wall assembly	4.3×10^-3^
cell wall assembly	5.0×10^-3^
cell differentiation	5.6×10^-3^
sporulation resulting in formation of a cellular spore	7.8×10^-3^
Reproduction	8.9×10^-3^
Sporulation	9.2×10^-3^

**Table 2:** **Predicted GO terms for 56 de novo genes** (**including 22 de novo genes with short promoters or poor alignments in promoters**) **by SPELL**[59]

**GO term**	***q-value***
M phase of meiotic cell cycle	8.5×10^-4^
Meiosis	8.5×10^-4^
meiotic cell cycle	1.0×10^-3^
cellular process involved in reproduction	1.0×10^-3^
reproductive process	1.0×10^-3^
Reproduction	3.1×10^-3^

## Discussion

We investigated the emergence of *cis*-regulatory elements in de novo genes. Specifically, 56 de novo genes were identified as having emerged in *S*. *cerevisiae* since separation from *S*. *paradoxus* approximately 5 million years ago [69]. It has been shown that different approaches for de novo gene identification may yield different results. For example, Capra et al. investigated all the de novo genes since WGD. This strategy ensured that the possibility of having orthologous genes in any species before WGD was avoided, but genes in the closely related species after WGD were allowed [38]. Wu et al., on the other hand, considered only the de novo genes without any orthologous genes but with highly similar orthologous regions and frame-shifts in two closely related species [27]. In short, Capra et al. discuss the evolution of de novo genes in a relative large time-scale while Wu et al. analyzed the characteristics of de novo genes that originated immediately by one-step mutations from closely related species. In this study, we attempted to understand the evolution of regulatory elements which requires sufficient evolution time to accumulate mutations. Therefore, we considered a time-scale that fell between the time-scales of the above two studies. We did not focus on the de novo genes that immediately emerged one-step away from non-coding regions as in Wu et al., because the promoters of these genes might not have experienced sufficient evolution time.

Our results showed that the promoters of new genes (of both de novo and duplicated origin) possessed similar numbers of regulatory TFs and TFBSs compared with those in orthologous genes. This finding suggested that TFBSs might be established rapidly after the emergence of a new gene and could be explained by the frequent occurrence of TFBS turnover, a well-documented phenomenon in eukaryote *cis*-regulation [34]. For example, frequent TFBS gain events in duplicated genes were found to play a critical role in the regulatory evolution of the yeast genome [12]. Papp et al. found that the numbers of TFBSs in the promoters of duplicated genes remained constant over evolutionary time, whereas the numbers of shared motifs from a preexisting gene decreased, perhaps because of a balance between the gain of new TFBSs and the loss of TFBSs from parent genes [12].

The promoters of de novo genes that evolved from non-coding regions instead of duplicated from promoters of parent genes might be expected to have a different frequency of TFBS gain event than in duplicated genes. However, our analyses showed that the de novo and duplicated new genes exhibited similar numbers of TFBS gain events. A simple explanation could be that preexisting TFBSs in the promoters of the de novo genes were more plentiful than previous expected. Indeed, our results indicated that more than half of the TFBSs in the promoters of de novo genes were preexisting TFBSs, which supports this explanation. Together with the observation of high substitution rates in the promoters of de novo genes, our results further suggested that the promoters experienced adaptation evolution and frequent gain events. Both these phenomena would rapidly increase the number of TFBSs in de novo genes to a level comparable with the number found in orthologous genes. In addition, the higher substitution rates in the promoters of de novo genes compared with those of neutral sequences (i.e. the four-fold degenerate sites) suggested that the new genes might experience positive selection during the establishment of *cis*-regulatory motifs. Our results agree with a previous protein interaction networks study which found that, although de novo genes initially had fewer functions and protein interactions than duplicated new genes, de novo genes rapidly gained functions and protein interactions until the numbers were comparable to duplicated new genes [38].

Research has shown that duplicated genes often inherit *cis*-regulatory elements from their parent genes, thereby benefiting from preexisting regulatory mechanisms [35,36]. However, because we found that de novo genes had a similar proportion of preexisting TFBSs in their promoters as duplicated new genes, we have proposed three possible explanations for this observation. First, studies have shown that non-functional TFBSs reside throughout the intergenic regions in the genome; for example, it was reported that TFs can bind to substantial numbers of non-functional TFBSs regardless of their weak binding strength [70]. Second, although we removed head-to-head genes that share core promoters, there still might be cases in which the promoters are shared. The promoter of the de novo genes may partially overlap with the distal promoter of neighboring genes, especially in yeast, which have relatively short intergenic region. Moreover, while non-functional TFBSs determined by documented regulatory associations in YEASTRACT have been removed (i.e. the pair of head-to-head genes would not have exactly the same set of TFBSs), some TFBSs may still be shared. These shared TFBSs could explain the unexpectedly high proportion of preexisting TFBSs in de novo genes. Third, there may be a number of false positives in the computational identification of the TFBSs [71]. Although we filtered out non-functional TFBSs in *S*. *cerevisiae* according to the regulatory associations documented in the YEASTRACT database [57], similar information in the other yeast species is insufficient to eliminate all the potential false positives. Thus, the numbers of TFBSs in other yeast species and consequently the number of preexisting TFBSs might have been overestimated.

The promoter architecture of new genes is an intriguing issue to explore because it has been associated with the gene origination mechanisms [38]. We found that duplicated new genes were enriched with OPN genes and TATA-containing genes; whereas, most de novo genes were TATA-less and enriched with DPN genes. The association between DPN and TATA-less promoters in de novo genes is consistent with the report that TATA-less promoters usually have clearer nucleosome free regions than TATA-containing genes [45,72]. Additionally, TATA box and OPN enrichment has been reported in the promoters of duplicated genes [44,73]. OPN and TATA-containing genes are relatively adaptable to environmental changes and are associated with processes that require high expression variation, such as transcriptional plasticity, sensitivity to chromatin regulation and genetic perturbations, expression noise, and expression divergence. In addition, TATA-containing genes are often highly regulated and are associated with inducible responses to stress or biotic stimuli [45,62,63,74]. DPN and TATA-less genes, on the other hand, display relatively low expression variation and constitutive expression, and TATA-less genes are lightly regulated by chromatin regulators, unresponsive to stress, and related to basic housekeeping functions in yeast and human [62,63,75]. The functions of TATA-less genes are enriched in basic processes such as cell growth and maintenance, protein biosynthesis, large ribosomal subunit, and mitochondrion [75], and these known functions are consistent with the results of our functional analyses of de novo genes. Furthermore, the promoters of the TATA-containing genes are TAF-independent and dominated by the Spt-Ada-Gcn5 acetyltransferase complex (SAGA), while the promoters of the TATA-less genes are TFIID-dominated and highly TAF-dependent despite there being a common set of TAFs that are shared by SAGA and TFIID [76]. As a result, the difference in TATA enrichment and nucleosome occupancy (OPN or DPN) between the two types of new genes indicates that they employ distinct regulatory mechanisms. These findings agree with the suggestions by Capra et al. that the function and fate of new genes are associated with their origins [38]. Our functional analysis using SPELL suggested that de novo genes might contribute to cellular processes that are involved in reproduction, such as sporulation and formations of cellular spore and cell wall. Differences in sporulation patterns and sporulation efficiencies between *S*. *cerevisiae* and *S*. *paradoxus* have been observed [77]. Also, germinating spores of *S*. *cerevisiae* show a higher preference for own-species mating than the spores of *S*. *paradoxus*[78]. In addition, the enrichment of DPN genes and TATA-less genes that we found in the de novo genes agrees with the observation that the genes involved in sporulation and division are constitutively expressed [79].

We used SPELL to predict the functions of de novo genes because of the lack of functional annotations in de novo genes. However, SPELL has various limitations. Given a set of query genes, SPELL identifies the expression microarray datasets that are most informative for these genes. Then additional genes that have the most similar expression profiles to the query genes are identified in the datasets. According to the functions of the additional genes, SPELL generates hypothetical functions for the query genes. However, the assignment of the functions is for the most part limited to the microarray datasets and GO annotation. Moreover, correlations of the expression patterns among a set of co-functional genes might not always be significantly high, because the genes need not be co-expressed at all the experimental time points. Because of these limitations, the functions assigned by SPELL may reveal only partial, and sometimes inaccurate, roles of de novo genes.

In addition to the SPELL functional predictions, we provided further support for the predicted de novo gene function by examining the function of their TFs. We identified BAS1, GCN4 and GCR1 as regulators of de novo genes. Interestingly, studies suggests that all three of these TFs are related to meiotic recombination, a process in reproduction: mutations in BAS1 affect the frequency of aberrant segregation of recombination hotspot at the histone HIS4 locus, lessen the recombination distance, and alter the frequency of meiosis-specific double-strand DNA breaks [65,66]; deletion or constitutive expression of GCN4 affects the frequency of gene conversion and crossing-over at the HIS4 locus [67]; and removal of GCR1-binding sites reduces the expression of REC102, a gene required for the initiation of meiotic recombination [68]. Based on previous studies and the findings in this study, we propose that de novo genes may play an important role in reproduction.

Although the functions of most de novo genes have not been well investigated, some of their specific roles have been addressed [1,2,27]. For example, Wu et al. have analyzed the transcriptome of numerous human tissues and found that de novo genes are highly expressed in the testes and cerebral cortex, which plays key roles in cognitive abilities [27]. The authors suggested that the de novo genes might contribute to phenotypic traits that are unique to humans [27]. Our results also suggest that new genes from different origins may play distinct roles in the evolutionary process. While duplicated new genes have been shown to be involved in environmental adaptation [38], we hypothesized that de novo genes might contribute to evolutionary innovation in reproduction processes like sporulation efficiency. Further studies are required to examine this hypothesis; nevertheless, the computational approaches that were used in this study shed some light on the evolution of *cis*-regulation in de novo genes.

## Conclusions

Our study showed that the number of TFBSs in de novo genes increased rapidly after gene emergence and soon resulted in that de novo genes having a comparable number of TFBSs as the orthologous genes. We suggested that frequent TFBS gain events, more numbers of unexpected preexisting TFBSs, and the lower selection pressure experienced in the promoters of de novo genes compared to orthologous genes could be the major reasons for this finding. Moreover, we found that new genes from different origins (de novo or duplication) have distinct regulatory characteristics (de novo genes were dominated by DPN and TATA-less genes; duplicated new genes were dominated by OPN and TATA-containing genes). Furthermore, we found that the predicted GO terms related to reproduction processes were enriched in de novo genes. Taking all of our results together, we concluded that de novo genes and duplicated new genes might play distinct roles in evolution.

## Competing interests

The authors declare no competing interests.

## Authors' contributions

ZTYT, HKT and DW designed the analyses. ZTYT, CHL and YFT collected the data. ZTYT performed the analyses. ZTYT, HKT, JHC and DW wrote the paper. HKT and DW were the principal investigators and conceived the analyses. All authors read and approved the final manuscript.

## Supplementary Material

Additional file 1 **Table S1.** The lists of de novo genes, duplicated new genes, and orthologous genes used in this study. Click here for file

Additional file 2 **Supplemental Document.** The document provides data and analysis in support of the main text, attempting to demonstrate that our findings are robust to various criteria of TFBS identifications. Click here for file
