# Supplementary material for: Evolution of cis-regulatory elements in yeast de novo and duplicated new genes
Source: BMC Genomics. 2012 Dec 21;13:717. doi: 10.1186/1471-2164-13-717 (PMC3553024; doi:10.1186/1471-2164-13-717)
Supplement: Additional file 2 — Supplemental Document. The document provides data and analysis in support of the main text, attempting to demonstrate that our findings are robust to various criteria of TFBS identifications. [file 1471-2164-13-717-S2.docx]

**Supplemental Document**

The document provides data and analysis in support of the main text, attempting to demonstrate that our findings are robust to various criteria of TFBS identifications.

# Methods

To further analyze the unexpected finding that the numbers of TFs and TFBSs are similar in de novo genes, duplicated new genes, and orthologous genes, we conducted a series of investigations on the evolution of TFBSs in attempt to explain the phenomena. These investigations depend on the identification of TFBSs; therefore, we re-identified TFBS with different criteria to examine the robustness of our findings. Briefly, the TFBS identification process is as follows: (a) obtain PWM from the MYBS database, (b) calculate cutoff of PWM score by PATSER, (c) scan PWM to find potential TFBSs, and (d) verify TFBSs by reliable regulatory relationships from YEASTRACT database. The most crucial step of TFBS identification is the determination of cutoff for each PWM. In the main text, we used the cutoff with *p*-value < 0.001 as suggested in [1]. Here, we repeated the investigation of TFBS characteristics using different sets of TFBSs under different criteria to demonstrate the robustness of our findings. We utilized two criteria: (a) cut-offs with *p*-value <0.0001 which are more stringent than that in main text (*p*-value <0.001), (b) cut-offs with PATSER default setting, which considers the motif length and information content of PWM, but the cut-off would not provide results under the same threshold of *p-*value. The first criterion is more stringent than that in main text. However under the second criteria, the strength of TFBS identification for each PWM would be different; therefore the TFBS dataset would be more biased and could be used to examine the robustness of our findings. The results are shown in the following section, and support the robustness of our findings: First, the new genes (both de novo and duplicated new genes) have similar numbers of TFs and TFBSs as orthologous genes. Next, the proportions of newly gained TFBSs are significantly higher in new genes than in orthologous genes. Last, the proportions of newly gained TFBSs are not significantly different between the two types of new genes.

# Results

## TFBS cut-off with *p*-value <0.0001

With *p*-value <0.0001, the results showed, on average, a de novo gene was regulated by 3.3 ± 0.5 TFs (11.5 ± 0.8 TFs in main text), a duplicated new gene was regulated by 3.7 ± 0.4 TFs (11.7 ± 0.6 TFs in main text), an orthologous gene was regulated by 4.0 ± 0.1 TFs (11.9 ± 0.3 TFs in main text). The average number of TFBSs identified in a promoter was 4.2 ± 0.7 (25.4 ± 1.9 in main text) for de novo genes, 4.3 ± 0.5 (24.4 ± 1.6 in main text) for duplicated new genes, and 4.9 ± 0.2 (24.3 ± 0.6 in main text) for orthologous genes. Although both the average numbers of TFs and TFBSs are smaller than the results in main text, the findings were not changed: The average numbers of TFs regulating a gene and the average number of TFBSs in a promoter were similar in the new genes (de novo or duplicated new) and old genes (orthologous) (Figure S1).

The numbers of TFBS gain events and the proportions of preexisting TFBSs of the TFBS dataset under different setting were also calculated. We obtained the same conclusions as main text. Significantly higher proportions of gained TFBSs were still observed for new genes (both de novo and duplicated new) as compared with orthologous genes (one-sided Wilcoxon test *p* = 8.3×10^-4^ and *p* = 3.7×10^-5^, respectively) (Figure S2). In average, 51.1% (53.2% in main text) and 46.5% (41.9% in main text) of TFBSs were found to be preexisting in the promoters of de novo genes and duplicated new genes, respectively; and 61.2% (65.3% in main text) were preexisting in the promoters of orthologous genes. The proportions of preexisting TFBSs were still significantly smaller in both de novo and duplicated new genes than orthologous genes (one-sided two-sample proportion test *p* = 2.7×10^-6^ and *p* = 5.3×10^-9^, respectively). In addition, no statistically significant difference was found between the two types of new genes (two-sided two-sample proportion test *p* = 0.09).

## TFBS cut-off using PATSER default setting

With PATSER default setting, the results showed, on average, a gene was regulated by 14.4 ± 1.0 TFs (11.5 ± 0.8 TFs in main text) in de novo genes, by 14.0 ± 1.1 TFs (11.7 ± 0.6 TFs in main text) in duplicated new genes, and by 15.8 ± 0.3 TFs (11.9 ± 0.3 TFs in main text) in orthologous genes. The number of TFBSs identified in a promoters was 30.2 ± 1.8 (25.4 ± 1.9 in main text) for de novo genes, 30.4 ± 2.2 (24.4 ± 1.6 in main text) for duplicated new genes, and 30.1 ± 0.5 (24.3 ± 0.6 in main text) for orthologous genes. Although both the average numbers of TFs and TFBSs are larger than results in main text, the findings were not changed: the average number of TFs regulating a gene and the average number of TFBSs in a promoter were similar between new genes (de novo or duplicated new) and old genes (orthologous) (Figure S3).

The numbers of TFBS gain events and the proportions of preexisting TFBSs of the TFBS dataset under different settings were also calculated. We obtained the same conclusions as main text. Significant higher proportions of gained TFBSs were still observed for new genes (de novo and duplicated new) compared with orthologous genes (one-sided Wilcoxon test *p* = 6.2×10^-4^ and *p* = 2.9×10^-5^, respectively) (Figure S4). In average, 55.9% (53.2% in main text) and 51.4% (41.9% in main text) of TFBSs were found to be preexisting in the promoters of de novo genes and duplicated new genes, respectively; and 65.8% (65.3% in main text) were preexisting in the promoters of orthologous genes. The proportions of preexisting TFBSs were still significantly smaller in both de novo and duplicated new genes than orthologous genes (one-sided two-sample proportion test *p* = 1.5×10^-11^ and *p* = 6.7×10^-12^, respectively). In addition, no statistically significant difference was found between the two types of new genes (two-sided two-sample proportion test *p* = 0.05).

# Reference

1. Turatsinze J-V, Thomas-Chollier M, Defrance M, van Helden J: **Using RSAT to scan genome sequences for transcription factor binding sites and *cis*-regulatory modules.** *Nature protocols* 2008, **3**:1578–88.

# Figures

**
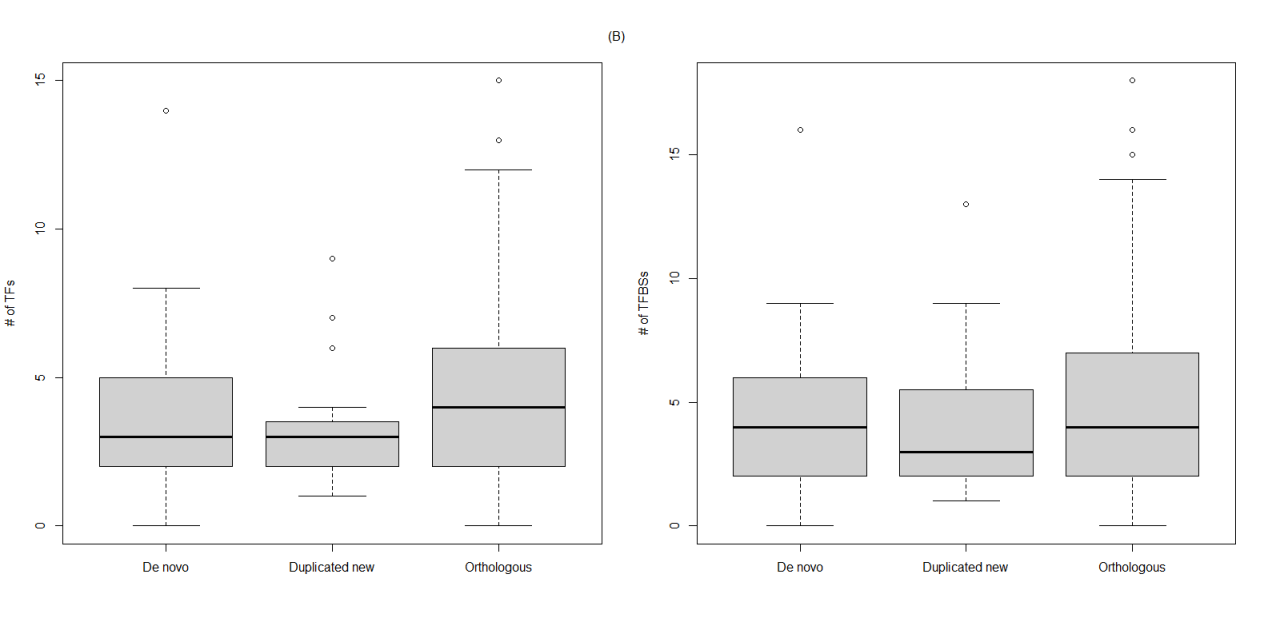
**

**Figure S1 - The numbers of TFs and TFBSs for de novo genes, duplicated new genes, and orthologous genes (TFBS cut-off with *p*-value < 0.0001).**

Comparison of the average number of TFs regulating a gene (A), and the average number of TFBSs in a gene (B) for de novo genes, duplicated new genes, and orthologous genes. No significant differences were observed (two-sided Wilcoxon test).

**
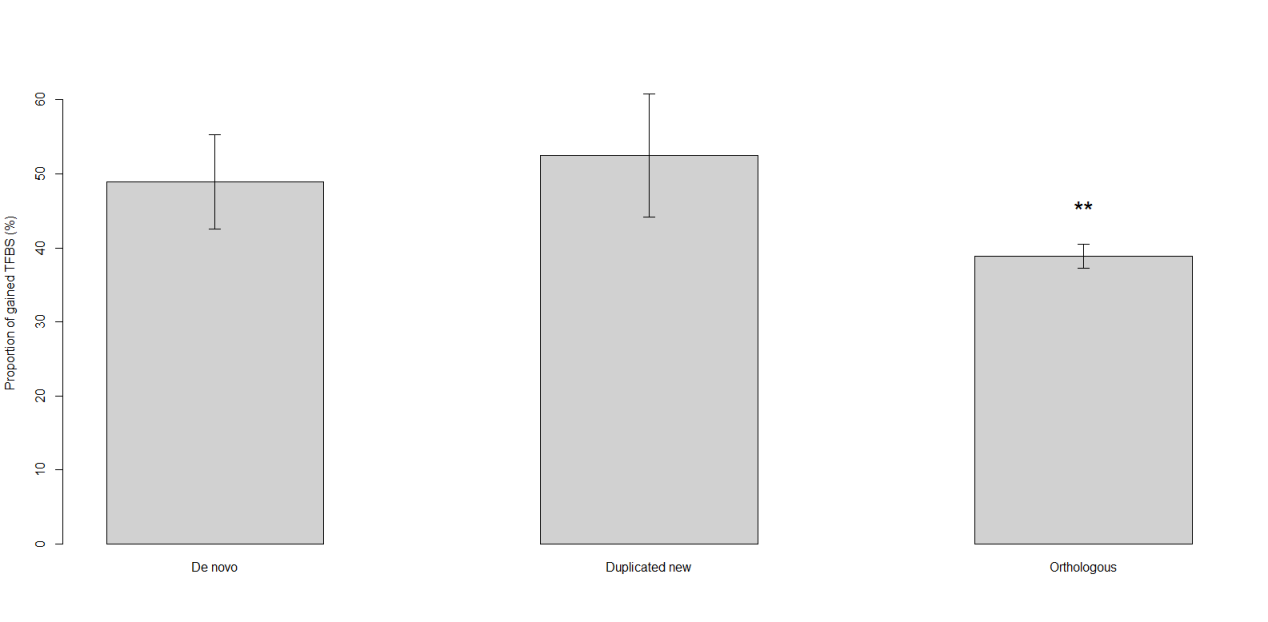
**

**Figure S2 - The proportions of gained TFBSs in de novo genes, duplicated new genes, and orthologous genes (TFBS cut-off with *p*-value < 0.0001).**

Comparisons of the proportions of gained TFBSs in de novo genes, duplicated new genes, and orthologous genes. The significance tests were conducted by one-sided Wilcoxon test (**: *p*-value < 0.001 compared with de novo genes).

**
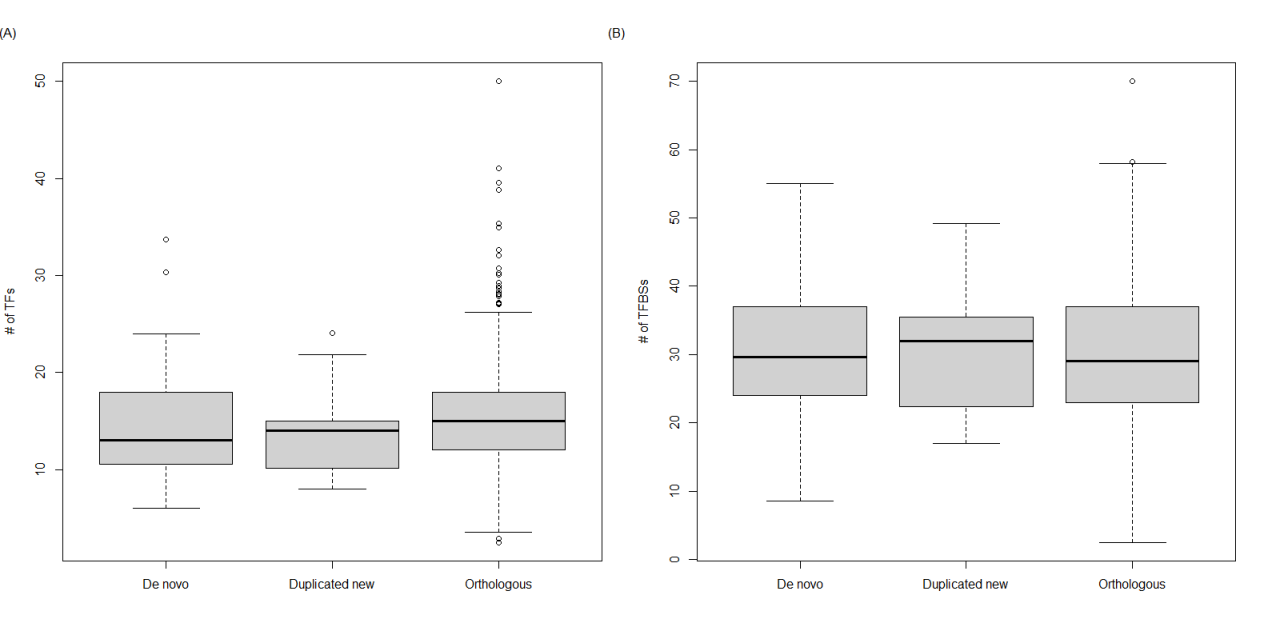
**

**Figure S3 - The numbers of TFs and TFBSs for de novo genes, duplicated new genes, and orthologous genes (TFBS cut-off using PATSER default setting).**

Comparison of the average number of TFs regulating a gene (A), and the average number of TFBSs in a gene (B) for de novo genes, duplicated new genes, and orthologous genes. No significant differences were observed (two-sided Wilcoxon test).

**
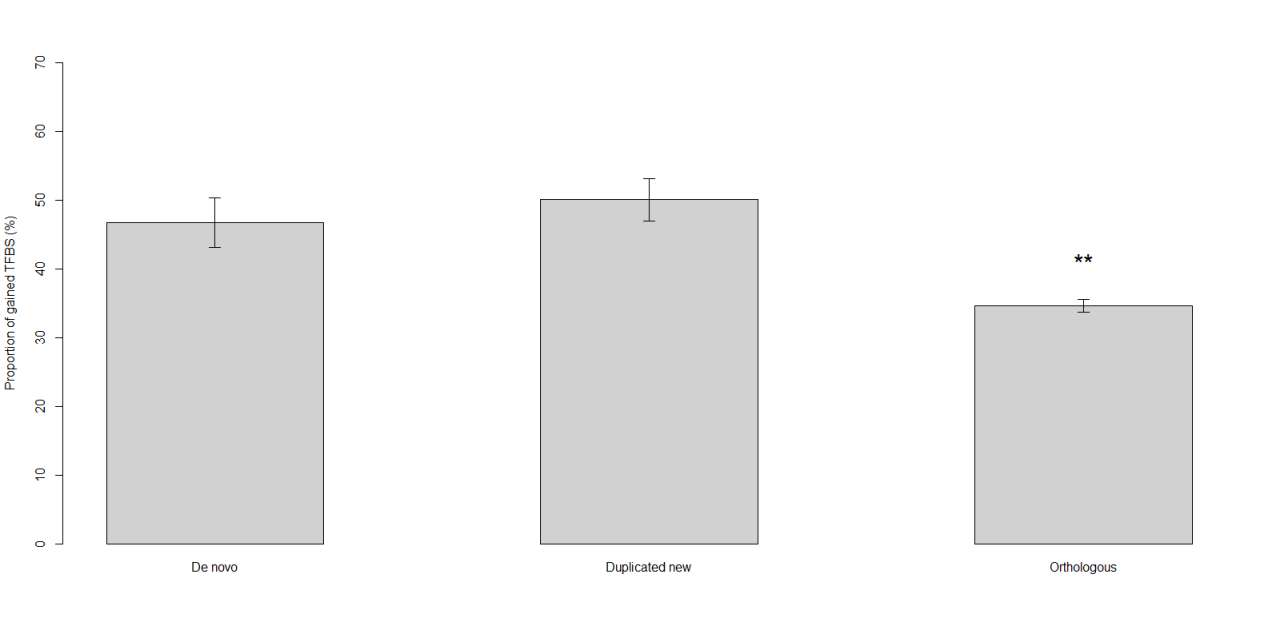
**

**Figure S4 - The proportions of gained TFBSs in de novo genes, duplicated new genes, and orthologous genes (TFBS cut-off using PATSER default setting).**

Comparisons of the proportions of gained TFBSs in de novo genes, duplicated new genes, and orthologous genes. The significance tests were conducted by one-sided Wilcoxon test (**: *p*-value < 0.001 compared with de novo genes).
